# Reduced kidney size and renal function of high-grade vesicoureteral reflux and intrarenal reflux in contrast-enhanced voiding urosonography

**DOI:** 10.3389/fped.2024.1478436

**Published:** 2024-12-18

**Authors:** Hualin Yan, Cong Wu, Jiehong Zhou, Cairong Huang, Xue Ma, Yidong Huang, Lugang Huang, Juxian Liu

**Affiliations:** ^1^ Department of Medical Ultrasound, West China Hospital, Sichuan University, Chengdu, China; ^2^ Department of Pediatric Surgery, West China Hospital, Sichuan University, Chengdu, China

**Keywords:** vesicoureteral reflux (VUR), intrarenal reflux (IRR), ultrasound, renal function, pediatric nephropathy

## Abstract

**Background:**

Vesicoureteral reflux (VUR) is a common pediatric urological condition associated with renal scarring, hypertension, and chronic kidney disease. Contrast-enhanced voiding urosonography (ceVUS) has emerged as a promising technique for diagnosing and evaluating VUR, with intrarenal reflux (IRR) often detected using this method. This study aimed to explore the relationship between different VUR grades and IRR on ceVUS, and assess the impact of VUR and IRR on kidney size and function.

**Methods:**

We reviewed all ceVUS studies from January 2019 to December 2023 conducted at West China Hospital, Sichuan University. Both video clips and digital images of the ceVUS examinations were recorded. A total of 220 uretero-renal units (URUs) of 110 children (67 males and 43 females) were included in this study.

**Results:**

Among the 220 URUs assessed, 134 were diagnosed with VUR, and 25 exhibited IRR, with IRR exclusively observed in patients with grade II VUR or higher. Upon age and sex matching, the severity of IRR showed a significant positive correlation with high-grade VUR (*P <* 0.001). Notably, patients with high-grade VUR and IRR displayed reduced kidney size compared to those without VUR or IRR (*P <* 0.05). Furthermore, patients with high-grade VUR and IRR had reduced DMSA renal function (*P* = 0.015, *P* = 0.012, respectively), and patients with high-grade VUR had more DMSA scars (*P* = 0.027), compared with those without VUR or IRR.

**Conclusion:**

Our study highlights that on ceVUS, the IRR degree was associated with the high-grade VUR, along with reductions in kidney size and renal function in patients with high-grade VUR and IRR.

## 1 Introduction

Vesicoureteral reflux (VUR) is a prevalent pediatric urological disorder characterized by the retrograde flow of urine from the bladder into the ureters and kidneys, with an incidence of nearly 1% (1). VUR poses an increased risk of urinary tract infections (UTIs), renal scarring, hypertension, and chronic kidney disease (CKD). In children with UTI or febrile UTI (fUTI), the incidence of VUR is approximately 30%, particularly affecting boys (1, 2). Following the first fUTI, renal ultrasonography is recommended to assess renal parenchymal damage and other urinary tract abnormalities (2, 3). Subsequent to recurrent UTIs or abnormal renal ultrasound findings, voiding cystourethrography (VCUG) is indicated for VUR detection (1, 2).

In recent years, contrast-enhanced voiding urosonography (ceVUS) has emerged as a promising technique for VUR diagnosis and assessment. ceVUS presents advantages over VCUG, including avoiding radiation exposure, non-invasiveness, and real-time imaging capabilities. Studies have demonstrated ceVUS's high sensitivity and specificity in detecting VUR, with high agreement in VUR grading between ceVUS and VCUG (4–6). Furthermore, compared to VCUG, ceVUS achieves a higher intrarenal reflux (IRR) detection rate by real-time urinary tract imaging (4). IRR, often overlooked, can lead to recurrent pyelonephritis, kidney scarring, and hypertension, affecting childhood renal development (7). Studies have indicated that IRR sites on ceVUS correspond to photon defect sites in technetium-99m-dimercaptosuccinic acid (^99m^Tc-DMSA) renal scans and parenchymal damage (8, 9).

Regarding kidney size and growth rate in childhood, Guarino et al. (10) found that a different length between the two kidneys in patients with VUR indicated an abnormal DMSA scan. However, there is limited research on the impact of different VUR grades and IRR on kidney size compared to normal kidneys in children of the same age and sex.

Therefore, we conducted this retrospective study to investigate clinical factors among different VUR grades and IRR on ceVUS, explore the relationship between different VUR grades and IRR on ceVUS, and assess the influence of VUR and IRR on kidney size and function. Our aim is to clarify the role of ceVUS in pediatric VUR management in clinical practice.

## 2 Materials and methods

### 2.1 Patients

This retrospective study was approved by the institutional review board of West China Hospital, Sichuan University (Trial Number: HX-G20-349). We reviewed all ceVUS studies from January 2019 to December 2023 conducted at West China Hospital, Sichuan University. The inclusion criteria were as follows: suspicion of VUR due to recurrent fUTIs or one fUTI combined with irregularity of ultrasonographic finding of the urinary tract. Patients with incomplete ceVUS videos or examination data were excluded. Following the ceVUS diagnostic criteria for VUR (grades I–V) and IRR (11, 12), a total of 220 uretero-renal units (URUs) of 110 children (67 males and 43 females) were included in this study (Table 1).

**Table 1:** Baseline characteristics of the patients and ceVUS results of uretero-renal units.

Characteristics	Value
Age months^a^	32.6 (8.6, 57.0)
Sex (male: female)^b^	67:43
ANH history^b^	5/110
Average fUTI times per year^c^	3.1 ± 2.9
VUR^d^	134/220
VUR negative	86
VUR Ⅰ	48
VUR Ⅱ	12
VUR Ⅲ	34
VUR Ⅳ	22
VUR Ⅴ	18
IRR^d^	25/220
IRR negative	195
Unilateral IRR	13
Bilateral IRR	12
Presence of hydronephrosis^d^	39/220
Grade of hydronephrosis^d^	
UTD-P1	21
UTD-P2	7
UTD-P3	11

ANH, antenatal hydronephrosis; fUTI, febrile urinary tract infection; IRR, intrarenal reflux; UTD, urinary tract dilation; VUR, vesicoureteral reflux.

^a^ Data are median with quartile 1 and quartile 3 in parentheses.

^b^ Data are numbers of patients.

^c^ Data are presented as mean ± standard deviation.

^d^ Data are the number of uretero-renal units.

### 2.2 The ceVUS examination

The Philips iU22 US system (Bothell, WA, USA) with an C5-1 convex array transducer (1-5 MHz) was employed. We used SonoVue® (Bracco, Italy) as the contrast agent, prepared by a pediatric nurse according to the manufacturer's instructions. A pediatric radiologist with over 10 years of experience in pediatric ultrasound (J.L.) performed the examination and diagnosis.

The examination procedures are briefly as follows: the bilateral kidneys, ureters, and bladder greyscale sonography are routinely performed before ceVUS examination. The kidney length, width, and thickness (anteroposterior diameter) are all measured. Written informed consent was obtained from the patient's parents or legal guardians. A urinary catheter was aseptically placed by the pediatric nurse before the examination, and the bladder was emptied by clamping the catheter. The patient, without sedation, assumed a supine position. Bladder capacity was calculated using the formula: volume = (age + 2) × 30 ml, with age in years. A contrast solution containing 0.5 ml of SonoVue® in 250 ml saline was instilled into the bladder under a pressure of 70 mmHg. The enhancement of the bilateral ureters, renal pelvis, renal calyces, and renal parenchyma, as well as their morphology, are stored in video clips and digital images. IRR was also record during the examination.

**Figure 1:**
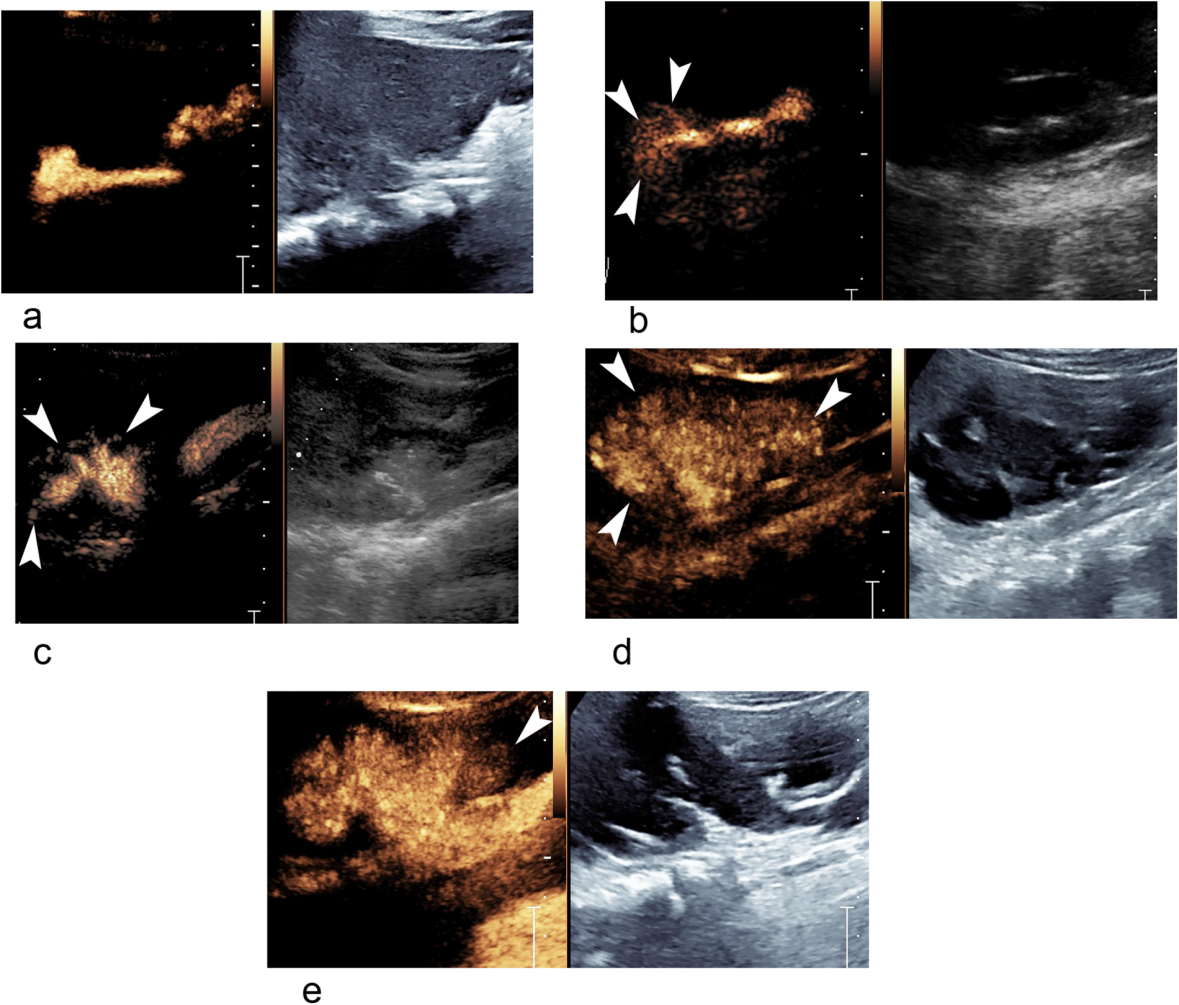
Different grades of VUR and IRR in ceVUS ultrasound images. **(a)** A 22-month-old boy with grade II VUR of right kidney without IRR. **(b)** A 6-month-old girl with grade II VUR and IRR (arrowheads) of right kidney. **(c)** A 5-month-old boy with grade III VUR and IRR (arrowheads) of right kidney. **(d)** A 13-month girl-old with grade V VUR and IRR (arrowheads) of right kidney. **(e)** The same patient as in **(d)**, grade IV VUR and IRR (arrowhead) of left kidney. IRR, intrarenal reflux; ceVUS, contrast-enhanced voiding urosonography; VUR, vesicoureteral reflux.

VUR was graded according to established guidelines (12) as follows: grade I, grade II, grade III, grade IV, grade V (Figure 1). Further, VUR was categorized into high-grade group (grade III–V) and low-grade group (grade I–II), while IRR was classified into negative, unilateral, and bilateral groups. Urinary tract dilation (UTD) grade was assessed using the UTD classification system (13). All final diagnoses were determined by joint video and image readings by two experienced pediatric radiologists (H.Y. and J.L.).

### 2.3 DMSA scan

Twenty patients from the study underwent a DMSA scan following the guidelines (14). Briefly, patients received intravenous injection of a radiopharmaceutical containing ^99m^Tc-DMSA, with radioisotope doses tailored to the patient's body surface area. Following administration, the radiopharmaceutical distributed and accumulated in the renal cortex, where it selectively bound to tubular epithelial cells. Imaging was typically conducted 2–4 h post-injection to allow for adequate uptake. Planar images of the kidneys were acquired using a gamma camera equipped with a low-energy, high-resolution collimator. Anterior and posterior views were obtained, with additional oblique or lateral views as deemed necessary. Static imaging was performed for 5–10 min per view. Subsequently, two independent radiologists interpreted the images to identify any focal or diffuse abnormalities indicative of renal cortical function and parenchymal scar. Split renal function (SRF) was evaluated based on four degrees: normal, slightly reduced, marked reduced, and no function.

### 2.4 Statistical analysis

Statistical analysis was conducted using SPSS v.25.0 software (IBM Corp., Armonk, NY, USA). Propensity score matching (PSM) for age and sex was employed to match different grade VUR groups with the VUR negative group, as well as to match different IRR groups with the IRR negative group. Continuous variables were compared using one-way analysis of variance (ANOVA) with Bonferroni's correction, while categorical variables were compared using the Chi-square test. Ranked variables were compared using the Mann–Whitney *U* test or the Kruskal–Wallis *H* test. Correlation analysis was performed using the Spearman correlation coefficient. *P* < 0.05 was considered statistically significant.

## 3 Results

### 3.1 Baseline characteristics

A total of 110 children (67 males and 43 females, median age 32.6 months) with 220 URUs were included in this study (Table 1). Five patients reported the ANH history. The average frequency of fUTI was 3.1 times per year. Among the URUs, the prevalence of VURs was 60.9% (134/220), as well as that the prevalence of IRR was 18.6% (25/134) among all URUs with VURs. Additionally, 39 URUs were found with UTD (Table 1).

### 3.2 Comparison among high-grade VUR, low-grade VUR and VUR negative groups

The high-grade VUR, low-grade VUR, and VUR negative groups were matched for age and sex, with no statistically significant differences between them (*P* = 0.828, 0.168, respectively, Table 2). Among all URUs with VUR positivity, no IRRs were observed in VUR grade I (as expected), while 8.33% (1/12) of IRRs were noted in VUR grade Ⅱ. In contrast, in the group with dilated VUR (grades Ⅲ, Ⅳ, and V), the prevalence of IRRs was 32.4% (24/74). This indicates that the prevalence of IRRs increases with the severity of VUR grade (Supplementary Table S1). The presence of IRR was significantly higher in the high-grade VUR patients, compared with the low-grade VUR and VUR negative groups (*P <* 0.001, Table 2 and Supplementary Table S1). The average fUTI times in the high-grade VUR group were more than observed in the low-grade VUR and VUR negative group (*P <* 0.001, Table 2 and Supplementary Table S2). Nevertheless, no notable discrepancy in the degree of hydronephrosis was discerned among high-grade VUR, low-grade VUR, and VUR negative groups (*P* = 0.637, Table 2). Notably, patients with high-grade VUR exhibited smaller kidney size, including length, width, and anteroposterior thickness, compared to those without VUR (*P* = 0.028, *P* = 0.033, and *P* = 0.014, respectively, Table 2 and Supplementary Table S2). However, there were no discernible differences in kidney dimensions between high-grade VUR and low-grade VUR groups or between low-grade VUR and VUR negative groups (Supplementary Table S2).

**Table 2:** Comparison among high-grade VUR, low-grade VUR and VUR negative groups.

	No VUR	Low grade VUR (Grade I–Ⅱ)	High grade VUR (Grade Ⅲ–V)	*P* value
Age/month^a^	13.0 (6.3, 36.6)	13.6 (7.7, 58.3)	15.4 (10.4, 58.5)	0.828
Sex (male: female)	41:14	42:18	44:30	0.168
Average fUTI times per year^b^	2.2 ± 1.7	2.5 ± 2.0	4.1 ± 3.2^c^	<0.001
Number of URUs	55	60	74	
IRR	0	1	24	<0.001
Grade of hydronephrosis (URUs)				0.637
UTD-P1	9	4	8	
UTD-P2	1	1	4	
UTD-P3	7	2	2	
Kidney size^b^				
Kidney length/cm	6.9 ± 1.4	6.7 ± 1.4	6.4 ± 1.3^c^	0.028
Kidney width/cm	3.3 ± 0.8	3.2 ± 0.6	3.0 ± 0.6^c^	0.033
Kidney anteroposterior thickness/cm	3.3 ± 0.7	3.2 ± 0.6	3.0 ± 0.6^c^	0.014

fUTI, febrile urinary tract infection; IRR, intrarenal reflux; URUs, uretero-renal units; UTD, urinary tract dilation; VUR, vesicoureteral reflux.

^a^ Data are median with quartile 1 and quartile 3 in parentheses.

^b^ Data are presented as mean ± standard deviation.

^c^ Multiple comparison was using Bonferroni's correction, which was shown in Supplementary Table S2.

### 3.3 Comparison among bilateral IRR, unilateral IRR and IRR negative groups

The bilateral IRR, unilateral IRR and IRR negative groups were matched for age and sex, with no statistically significant differences observed among them (*P* = 0.926, 0.955, respectively, Table 3). Of all IRRs identified in this study, 96% (24/25) were observed in dilating VUR (grades Ⅲ, Ⅳ, and V), while only 4% (1/25) were detected in non-dilating VUR. The bilateral IRR group had higher grade VUR compared to the unilateral IRR group (*P <* 0.001, Table 3). No significant disparity in the average frequency of fUTI was noted among the different IRR groups (*P* = 0.076, Table 3). Similarly, no significant difference in the degree of hydronephrosis was observed among the various IRR groups (*P* = 0.922, Table 3). Interestingly, both the kidney width in patients with bilateral IRR and unilateral IRR were smaller than those in patients without IRR (*P* = 0.006 and *P* = 0.007, respectively, Supplementary Table S3). Likewise, the anteroposterior thickness of kidneys in patients with unilateral IRR was smaller than those in patients without IRR (*P* = 0.017, Supplementary Table S3). However, no discernible difference in kidney length was identified among the different IRR groups (*P* = 0.21, Table 3).

**Table 3:** Comparison among bilateral IRR, unilateral IRR and IRR negative groups.

	No IRR	Unilateral IRR	Bilateral IRR	*P* Value
Age/month^a^	10.2 (6.2, 59.1)	12.6 (6.2, 41.8)	12.3 (5.0, 13.5)	0.926
Sex (male: female)	8:5	8:5	8:4	0.955
Average fUTI times per year^b^	1.8 ± 0.8	4.1 ± 3.1	2.4 ± 1.4	0.076
Number of URUs	13	13	12	
VUR grade (URUs)				
negative	13	0	0	<0.001
Ⅰ	0	0	0	
Ⅱ	0	1	0	
Ⅲ	0	1	4	
Ⅳ	0	4	6	
Ⅴ	0	7	2	
Grade of hydronephrosis (URUs)				0.922
UTD-P1	3	0	1	
UTD-P2	0	2	1	
UTD-P3	4	1	1	
Kidney size^b^				
Kidney length/cm	6.8 ± 1.6	5.9 ± 1.5	6.0 ± 0.7	0.210
Kidney width/cm	3.6 ± 0.9	2.6 ± 0.5	2.6 ± 0.5^c^	0.002
Kidney anteroposterior thickness/cm	3.5 ± 0.8	2.7 ± 0.8	2.8 ± 0.4^c^	0.015

fUTI, febrile urinary tract infection; IRR, intrarenal reflux; URUs, uretero-renal units; UTD, urinary tract dilation; VUR, vesicoureteral reflux.

^a^ Data are median with quartile 1 and quartile 3 in parentheses.

^b^ Data are presented as mean ± standard deviation.

^c^ Multiple comparison was using Bonferroni's correction, which was shown in Supplementary Table S3.

### 3.4 DMSA split renal function and scar

Split renal function (SRF) and DMSA scar were assessed using DMSA as previously described (Supplementary Table S4). The VUR grade significantly correlated with the DMSA SRF (ρ =  0.449), *P* = 0.004) and DMSA scar (ρ =  0.362, *P* = 0.025). Furthermore, higher-grade VUR was associated with reduced DMSA SRF and a greater number of DMSA scars compared to lower-grade VUR (*P* = 0.015, *P* = 0.027, respectively). Similarly, positive IRR significantly correlated with the DMSA SRF (ρ = 0.447, *P* = 0.004). Patients with IRR exhibited reduced DMSA SRF compared to those without IRR (*P* = 0.012). However, no significant correlation was observed between positive IRR and the DMSA scar (*P* = 0.114).

## 4 Discussion

In this retrospective study, 134 VUR and 25 IRR were detected by ceVUS among 220 URUs. Notably, IRR was exclusively detected in cases of grade II VUR or higher, with the severity of IRR being positively correlated with the high-grade VUR. fUTI occurred more frequently in patients with high-grade VUR and patients with IRR. Remarkably, patients with high-grade VUR and IRR exhibited a reduction in kidney size compared to those without VUR or IRR. Furthermore, patients with high-grade VUR and IRR had reduced DMSA renal function, and patients with high-grade VUR had more DMSA scars, compared with those without VUR or IRR.

High-grade VUR constitutes a significant risk factor for renal scarring and recurrent UTI (15, 16). According to the latest Asian or European guidelines (1, 17, 18), children with a fUTI and high-grade VUR can still be considered for initial medical treatment with continuous antibiotic prophylaxis (CAP), and surgical reimplantation should be considered in patients with high-grade VUR with recurrent breakthrough febrile UTI on antibiotic prophylaxis. In this study, we found that patients with high-grade VUR are associated with a shrunk kidney, and had reduced renal function and more kidney scarring. Our findings provide substantial evidence supporting the current therapeutic approach for high-grade VUR patients. The follow-up study of ceVUS after treatment is needed to assess treatment efficacy.

IRR refers to the retrograde flow of urine from the renal pelvis into the renal parenchyma, typically occurring in conjunction with compound renal papillae at the polar regions of the kidneys (19). Studies suggests that IRR, particularly when associated with infected urine, triggers inflammatory reactions, fibrosis of the renal parenchyma, and eventual renal scarring (20, 21). Nonetheless, the prognostic implications and treatment strategies for IRR remain contentious. Boubnova J. et al. reported that under medical management, the prognosis for IRR is not different from high-grade VUR without IRR (22). However, accumulating evidence demonstrates that, IRR sites on ceVUS corresponds with the sites of photon defects on DMSA renal scans (8, 9). IRR can lead to recurrent pyelonephritis and kidney scarring, and the VUR patients with IRR are more likely to present a decreased differential renal function and breakthrough UTI (7, 23). Xiuzhen Y. et al. also demonstrated the presence of IRR can impact renal growth in children diagnosed with Grades III-V primary VUR (24). Our study contributes to this body of knowledge by demonstrating reduced DMSA renal function and kidney size in patients with IRR, highlighting the potential hazard of IRR on pediatric renal development.

Because of the advantage of radiation-free and real-time imaging capabilities over VCUG, ceVUS can detect more IRR and IRR in low-grade VUR. In our study, the incidence of IRR waws 18.6% in patients with VUR, which was consistent with previous studies (25–27). Cvitkovic-Roic A (25). and Klein E.L. et al. (26) also reported that IRR on ceVUS was found in grade II VUR, consistent with our study findings. Given that children with IRR are prone to renal scarring and diminished renal function, whether the presence of IRR in low-grade VUR patients warrants alterations in current management guidelines? Further multicenter prospective studies are needed to answer such questions.

There are several limitations of this study. Firstly, only 20 patients underwent DMSA scan, and we did not analysis the relationship of IRR sites and the sites of photon defects on DMSA scans due to the limited data. DMSA only reflected the relative renal function, and we plan to perform Tc-99m diethylenetriaminepentaacetic acid (DTPA) in future studies to obtain more precise renal function data. Secondly, we did not conduct comparative study with VCUG. Thirdly, sometime VUR is secondary to posterior urethral valves that ceVUS is difficult to detect. The European Society of Paediatric Radiology still recommend VCUG as the gold standard to rule out posterior urethral valves and to study urethral anatomy (28). Finally, we did not follow up and ceVUS and the prognosis of patients with/without IRR after treatment in the present study. And we will conduct the follow-up study in the future.

In conclusion, our study highlights the association between the degree of IRR and high-grade VUR observed on ceVUS, along with reductions in kidney size and renal function in patients with high-grade VUR and IRR. This is a preliminary study of the VUR grade and IRR in ceVUS, we will continue the follow-up study after treatment and compare outcomes of different patients.

## Data Availability

The original contributions presented in the study are included in the article/Supplementary Material, further inquiries can be directed to the corresponding author.
