# Supplementary material for: Reduced kidney size and renal function of high-grade vesicoureteral reflux and intrarenal reflux in contrast-enhanced voiding urosonography
Source: Front Pediatr. 2024 Dec 18;12:1478436. doi: 10.3389/fped.2024.1478436 (PMC11688366; doi:10.3389/fped.2024.1478436)
Supplement: Supplementary file 1 [file Table1.docx]

Supplementary Materials

Table S1 The comparison of VUR grade among different VUR grades

|  | IRR degree | | | *P* Value |
| --- | --- | --- | --- | --- |
|  | No IRR | Unilateral IRR | Bilateral IRR |  |
| VUR grade |  |  |  |  |
| negative | 55 | 0 | 0 | ＜0.001 |
| Ⅰ | 48 | 0 | 0 |  |
| Ⅱ | 11 | 1 | 0 |  |
| Ⅲ | 29 | 1 | 4 |  |
| Ⅳ | 12 | 4 | 6 |  |
| Ⅴ | 9 | 7 | 2 |  |

*IRR* intrarenal reflux, *VUR* vesicoureteral reflux

Table S2 Multiple comparisons among different VUR groups

| Parameter | Multiple comparison | *P* Value^a^ |
| --- | --- | --- |
| Average fUTI times | High grade VUR vs. Low grade VUR | 0.006 |
|  | High grade VUR vs. No VUR | 0.001 |
|  | Low grade VUR vs. No VUR | 1.000 |
| Kidney length/cm | High grade VUR vs Low grade VUR | 0.255 |
|  | High grade VUR vs No VUR | 0.028 |
|  | Low grade VUR vs No VUR | 0.299 |
| Kidney width/cm | High grade VUR vs Low grade VUR | 0.318 |
|  | High grade VUR vs No VUR | 0.032 |
|  | Low grade VUR vs No VUR | 1.000 |
| Kidney anteroposterior thickness/cm | High grade VUR vs Low grade VUR | 0.170 |
|  | High grade VUR vs No VUR | 0.004 |
|  | Low grade VUR vs No VUR | 0.130 |

^a^Multiple comparison was using Bonferroni’s correction. *fUTI* febrile urinary tract infection, *IRR* intrarenal reflux, *VUR* vesicoureteral reflux

Table S3 Multiple comparisons among different IRR groups

| Parameter | Multiple comparison | *P* Value^a^ |
| --- | --- | --- |
| Kidney width/cm | Bilateral IRR vs. Unilateral IRR | 1.000 |
|  | Bilateral IRR vs. No IRR | 0.006 |
|  | Unilateral IRR vs. No IRR | 0.007 |
| Kidney anteroposterior thickness/cm | Bilateral IRR vs. Unilateral IRR | 1.000 |
|  | Bilateral IRR vs. No IRR | 0.093 |
|  | Unilateral IRR vs. No IRR | 0.017 |

^a^Multiple comparison was using Bonferroni’s correction. *IRR* intrarenal reflux

Table S4 Detail DMSA split renal function and scar results

| Patient No. | Sex | Age/ months | fUTI times | Left URU | | | | | Right URU | | | | |
| --- | --- | --- | --- | --- | --- | --- | --- | --- | --- | --- | --- | --- | --- |
|  |  |  |  | VUR grade | IRR | UTD | DMSA scar site | DMSA SRF | VUR grade | IRR | UTD | DMSA scar site | DMSA SRF |
| 1 | male | 5.0 | 2 | 4 | Yes | No | No | normal | 3 | Yes | No | No | normal |
| 2 | male | 89.4 | 0 | 0 | No | No | No | normal | 5 | Yes | P2 | No | marked reduced |
| 3 | male | 3.3 | 2 | 0 | No | P3 | Upper, middle and lower | marked reduced | 0 | No | No | No | normal |
| 4 | male | 10.2 | 2 | 0 | No | No | No | normal | 4 | No | No | No | marked reduced |
| 5 | female | 13.1 | 1 | 4 | Yes | P3 | No | slightly reduced | 5 | Yes | P2 | No | marked reduced |
| 6 | male | 8.6 | 1 | 0 | No | P1 | No | normal | 0 | No | No | No | normal |
| 7 | female | 42.3 | 4 | 0 | No | No | No | normal | 5 | No | No | Upper, middle and lower | 3 |
| 8 | male | 11.9 | 3 | 3 | No | No | Upper | marked reduced | 1 | No | No | No | normal |
| 9 | female | 59.6 | 8 | 5 | No | P1 | Lower | marked reduced | 3 | No | No | No | normal |
| 10 | female | 12.8 | 1 | 2 | No | No | No | normal | 3 | No | No | No | normal |
| 11 | female | 58.5 |  | 2 | No | No | No | normal | 1 | No | No | No | normal |
| 12 | female | 109.7 | 8 | 0 | No | No | No | slightly reduced | 0 | No | No | Upper | slightly reduced |
| 13 | male | 9.8 | 2 | 3 | No | No | No | marked reduced | 0 | No | No | No | normal |
| 14 | male | 40.6 | 3 | 5 | No | No | Upper, middle and lower | no function | 5 | No | No | Upper, middle and lower | no function |
| 15 | female | 77.9 |  | 3 | No | No | No | normal | 3 | No | No | No | normal |
| 16 | female | 70.2 | 10 | 3 | No | No | No | normal | 5 | Yes | No | No | marked reduced |
| 17 | male | 20.6 |  | 0 | No | No | No | normal | 0 | No | No | No | marked reduced |
| 18 | male | 101.5 | 4 | 4 | No | No | No | normal | 5 | No | No | Upper, middle and lower | no function |
| 19 | male | 23.0 | 1 | 1 | No | No | No | normal | 1 | No | No | No | normal |
| 20 | male | 19.3 | 6 | 1 | No | No | No | slightly reduced | 0 | No | No | No | normal |

*DMSA* technetium-99m-dimercaptosuccinic acid, *fUTI* febrile urinary tract infection, *IRR* intrarenal reflux, *SRF* split renal function, *URUs* uretero-renal units, *UTD*, urinary tract dilation, *VUR* vesicoureteral reflux
